# Low interspecific variation and no phylogenetic signal in additive genetic variance in wild bird and mammal populations

**DOI:** 10.1002/ece3.10693

**Published:** 2023-11-05

**Authors:** Euan A. Young, Erik Postma

**Affiliations:** ^1^ Groningen Institute for Evolutionary Life Sciences University of Groningen Groningen The Netherlands; ^2^ Centre for Ecology and Conservation University of Exeter Penryn UK

**Keywords:** evolvability, genetic variance, heritability, meta‐analysis, phylogenetic signal, quantitative genetics

## Abstract

Evolutionary adaptation through genetic change requires genetic variation and is a key mechanism enabling species to persist in changing environments. Although a substantial body of work has focused on understanding how and why additive genetic variance (*V*_A_) differs among traits *within* species, we still know little about how they vary *among* species. Here we make a first attempt at testing for interspecific variation in two complementary measures of *V*_A_ and the role of phylogeny in shaping this variation. To this end, we performed a phylogenetic comparative analysis using 1822 narrow‐sense heritability (*h* ^2^) for 68 species of birds and mammals and 378 coefficients of additive genetic variance (*CV*_A_) estimates for 23 species. Controlling for within‐species variation attributable to estimation method and trait type, we found some interspecific variation in *h* ^2^ (~15%) but not *CV*_A_. Although suggestive of interspecific variation in the importance of non‐(additive) genetic sources of variance, sample sizes were insufficient to test this hypothesis directly. Additionally, although power was low, no phylogenetic signal was detected for either measure. Hence, while this suggests interspecific variation in *V*_A_ is probably small, our understanding of interspecific variation in the adaptive potential of wild vertebrate populations is currently hampered by data limitations, a scarcity of *CV*_A_ estimates and a measure of their uncertainty in particular.

## Graphical abstract

Evolutionary adaptation through genetic change requires genetic variation, but little is known about how much this varies across species. To this end, we performed a phylogenetic comparative analysis using 1822 narrow‐sense heritability for 68 species of birds and mammals and 378 coefficients of additive genetic variance estimates for 23 species. Although data limitations meant statistical power was low, we found that species varied little in their additive genetic variance and none of this was explained by phylogeny.

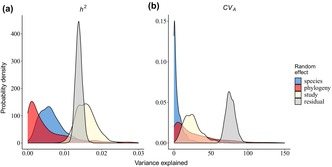

## 1 INTRODUCTION

Dispersal, plasticity and evolutionary (i.e. genetic) adaptation are three important mechanisms enabling organisms to persist in the face of environmental change (Gienapp & Brommer, 2014; Gonzalez et al., 2013; Lande & Shannon, 1996). Following classical quantitative genetic theory, evolutionary adaptation requires: (1) additive genetic variance (*V*_A_) in fitness; (2) *V*_A_ in the phenotype in question; and (3) a positive covariance between them (Lande, 1979; Lush, 1937; Lynch & Walsh, 1998). A recent study has demonstrated that substantial *V*_A_ in fitness exists across 19 wild bird and mammal populations (Bonnet et al., 2022). If representative, this suggests that birds and mammals are adapting to environmental change through natural selection. While it is also well established that variation in nearly all traits is underpinned to some degree by *V*_A_ (Hill et al., 2008), it is less clear whether *V*_A_ varies systematically across species. In a time of unprecedented levels of environmental change (Butchart et al., 2010; IPCC, 2013; Palumbi, 2001), understanding how species and populations vary in their ability to adapt to these changes is a key aim in evolutionary biology (Gienapp et al., 2008; Teplitsky et al., 2014; Visser, 2008).

A meaningful comparison of *V*_A_ across a variety of traits and species requires standardisation. One common standardisation is to divide the additive genetic variance by the total phenotypic variance to obtain the proportion of the phenotypic variance attributable to additive genetic effects, that is the narrow‐sense heritability (*h* ^2^) (Falconer & Mackay, 1996). In the absence of information on the selection differential, however, *h* ^2^ is a poor measure of a trait's evolvability, that is its expected response to selection (Houle, 1992). Furthermore, as *h* ^2^ is a measure of the amount of additive genetic variance relative to the total amount of phenotypic variance, a low *h* ^2^ is indicative of either low levels of additive genetic variance or high levels of other sources of variation. Instead, it has been suggested that the coefficient of additive genetic variance (*CV*_A_), a mean‐standardised measure of additive genetic variation, does not suffer from these limitations and hence provides a superior measure of a trait's evolvability (Garcia‐Gonzalez et al., 2012; Houle, 1992). However, *CV*_A_ remains far less commonly reported than *h* ^2^, among others, because it is only appropriate for specific traits (e.g. traits measured on a true ratio scale) (Pélabon et al., 2020).

After standardising *V*_A_ as *h* ^2^ or *CV*_A_, substantial intraspecific variation between estimates within species exists owing to systematic differences among trait types, which has previously been described and discussed in detail Mousseau and Roff (1987), Postma (2014), Stirling et al. (2002). Broadly speaking, traits that are more closely related to an individual's fitness (e.g. lifetime reproductive success, a commonly used proxy of an individual's fitness) have been shown to have lower estimates of *h* ^2^, but the fact that they do not have lower estimates of *CV*_A_ tells us that this is because of a larger role for non‐additive and/or environmental effects in shaping variation in fitness traits (McCleery et al., 2004; Teplitsky et al., 2009). In fact, morphological traits are generally found to have a lower C*V*_A_ than life‐history traits (Mittell et al., 2015; Mousseau & Roff, 1987; Postma, 2014; Price & Schluter, 1991). This is interpreted as evidence in support of the target size hypothesis, which predicts higher levels of *V*_A_ in traits more closely associated with fitness because they are assumed to be influenced by more genetic loci (Houle, 1991, 1992, 1998). Having said this, it remains less well understood how behavioural and physiological traits fit into this framework (Stirling et al., 2002). This is unfortunate, as behavioural changes are often the first line of defence against environmental change (Charmantier et al., 2008), and physiology is thought to be an important driver of life‐history evolution (Crespi et al., 2013).

Far fewer studies have examined differences in *V*_A_ among species (but see Dochtermann et al., 2019; Martinossi‐Allibert et al., 2017; Mittell et al., 2015; Wood et al., 2016). Aside from applications to conservation, studying interspecific variation in *V*_A_ can provide insight into the mechanisms shaping *V*_A_ and contribute to our understanding of its stability (Arnold et al., 2008). This, however, is not a trivial task, owing to the variety of mechanisms by which interspecific variation may be generated (recently reviewed in Pélabon et al., 2023). While species with larger populations are expected to have higher levels of *V*_A_ (Hill, 1982), ultimately this will depend upon both the historical population sizes (e.g. genetic bottlenecks would reduce *V*_A_) and the degree of gene flow both within and from outside the population (Lande, 1988). Selection also shapes *V*_A_ in complex ways: While stabilising selection is expected to erode *V*_A_ (Fisher, 1930), if this is heterogeneous across small (spatial or temporal) scales, it can increase *V*_A_ through promoting different polymorphisms (Hedrick et al., 1976). Furthermore, single‐species studies have generated a wide variety of hypotheses relating to how environmental conditions may affect both *h* ^2^ and *V*_A_ (reviewed in Hoffman & Merilä, 1999). In studies of wild populations, for example, unfavourable conditions lead to lower *h* ^2^ owing to a relatively lower importance of genes compared with factors such as parental care or habitat quality (Charmantier & Garant, 2005; Gebhardt‐Henrich & Van Noordwijk, 1991). However, examining any one of these hypotheses across species opens an array of methodological issues (e.g. how would one standardise a measure of environmental harshness across species?). Indeed, these complexities are one explanation for why Wood et al. (2016) found no evidence for *h* ^2^ to covary with adult census population size or the strength, direction or form of selection across 83 species.

Instead, here we take a step back: Rather than searching for population‐ or species‐specific correlates of *h* ^2^ or *CV*_A_, we quantify interspecific variation in *V*_A_ and how much of this variation correlates with phylogeny. This provides an estimate of the *opportunity* for species‐specific properties to shape absolute and relative levels of *V*_A_, some of which may be more similar among closely related species. For example, if environmental conditions shape *h* ^2^ and/or *CV*_A_ (Charmantier & Garant, 2005; Gebhardt‐Henrich & Van Noordwijk, 1991) and more closely related species occupy more similar environments (Blomberg & Garland, 2002; Losos, 2008), this would give rise to a phylogenetic signal in measures of *V*_A_.

To our knowledge, only two studies have explicitly tested for a phylogenetic signal in *V*_A_ across traits (Dochtermann et al., 2019; Martinossi‐Allibert et al., 2017). Dochtermann et al. (2019) found no phylogenetic signal, but it was limited to behavioural traits, and Martinossi‐Allibert et al. (2017) showed a small phylogenetic signal in *V*_A_ but did not allow for other sources of interspecific variation in their model, which may have upwardly biased their estimate of the importance of phylogeny. Additionally, both studies primarily used the phylogeny to control for potential non‐independence of species estimates while estimating other fixed and random effects of interest (Felsenstein, 1985; Nakagawa & Santos, 2012) and do not discuss interspecific variation (or its absence) in any detail.

Here, we present a phylogenetic comparative analysis of univariate estimates of *h* ^2^ and *CV*_A_ to quantify interspecific variation in *V*_A_ in the wild. To do so, we used 1822 *h* ^2^ and 378 *CV*_A_ estimates for, respectively, 68 and 23 species of birds and mammals obtained in the wild, while controlling for methodological and trait differences. For the latter, we classify traits as either behaviour, life history, morphology, physiology or fitness traits (measures of lifetime reproductive success). After controlling for these differences, we then present the level of interspecific *V*_A_ according to each metric and to what extent it can be explained by phylogenetic relatedness.

## 2 METHODS

### 2.1 Literature search and criteria

We used a collection of published estimates of variance‐ and mean‐standardised additive genetic variance (narrow‐sense heritability (*h* ^2^) and coefficients of additive genetic variance (*CV*_A_), respectively) for wild populations of birds and mammals previously used in Postma (2014), covering studies published until 2012, updated to include studies published up to April 2020. Studies included in the original dataset were identified using the Web of Science database (https://apps.webofknowledge.com) with the following search terms in the ‘Topic’ field: (‘wild population*’ OR ‘natural population*’) AND (‘heritabil*’ OR ‘genetic* estimate*’). Additionally, estimates from all studies cited in Merilä et al. (2001) and all publications that cited Kruuk (2004), Wilson et al. (2010) and Hadfield (2010) were included. These were complemented with estimates from studies published after 2012 that cited Wilson et al. (2010). As our primary goal was to gain insight into genetic variance in the wild, we included only studies carried out in a relevant ecological setting involving no breeding manipulations. We limited ourselves to birds and mammals, as they are the most widely studied taxa in the wild and the only two taxa for which the number of species was sufficient for the analyses outlined below.

### 2.2 Quantifying additive genetic variance

Whenever possible, we recorded all reported estimates of *h* ^2^ and *CV*_A_. This includes estimates for the same trait but from different models (e.g. with different combinations of fixed effects, different covariates or single vs. multivariate models), unless the authors explicitly stated that they considered one estimate superior.

If *h* ^2^ was not provided, it was calculated from the additive genetic variance (*V*_A_) and phenotypic variance (*V*_P_) reported by the authors as:

$$h^{2}=\frac{V_{A}}{V_{P}}$$

Note that whether this estimate of *V*_P_ is conditioned on variation attributable to one or more fixed effects will vary among studies (Wilson, 2008).

If not provided by the authors, *CV*_A_ was calculated as a percentage from *V*_A_ and the trait mean ($\bar{x}$) following Garcia‐Gonzalez et al. (2012):

$$CV_{A}=100\times \frac{\sqrt{V_{A}}}{\bar{x}}$$

Estimates of *CV*_A_ were only included for traits on a true ratio scale (Pélabon et al., 2020), and no log‐transformed trait estimates were included. Estimates of *I*_A_ (defined as $V_{A}/{\bar{x}}^{2}$; Houle, 1992) were converted to *CV*_A_ by taking the square root of *I*_A_ multiplied by 100. Standard errors (SEs) were recorded as a measure of the precision of estimates used for weighting estimates in the meta‐analytic mixed models outlined below. If only 95% confidence or credible intervals were available, approximate SEs were calculated from these by dividing half the difference between the upper and lower 95% intervals by 1.96. As *CV*_A_ estimates are rarely accompanied by an estimate of their precision (Garcia‐Gonzalez et al., 2012), we approximated their SE from the SE accompanying the estimate of *h* ^2^ for that trait by assuming the z‐value (estimate divided by SE) is the same for both.

### 2.3 Predictor variables

Previous analyses have suggested that larger sample sizes result in lower (less inflated) *h* ^2^ estimates (Postma, 2014). To control for this, sample sizes were recorded as the number of individual phenotypes used in the analysis, discounting multiple measurements of the same individual.

We classified traits as morphology, life history, fitness, physiology or behaviour traits. We acknowledge that these classifications are, to some degree, arbitrary and that some traits could be classified in multiple ways. In these instances, we followed the classification used in the original publication. In particular, we treat fitness traits as separate from other life histories, as traits more closely related to fitness are predicted to have different levels of genetic variance (Houle et al., 1996). Fitness traits included any measure of an individual's lifetime reproductive success (Brommer et al., 2004, 2007; Hayward et al., 2018).

The publication year was recorded to account for temporal changes in the size of estimates, thought to be largely due to methodological differences (Postma, 2014). Consequently, the specific method used to estimate *V*_A_ and the derived parameters *h* ^2^ and *CV*_A_ were also recorded. The classes of methods used for this were: parent–offspring regression, animal model (MCMC) (i.e. Bayesian), animal model (REML), grandparent–offspring regression, half‐sib and full‐sib analyses.

Each study was given its own unique ID to allow us to model any non‐independence among estimates for different traits from the same study.

### 2.4 Statistical analyses

We examined how *h* ^2^ and *CV*_A_ vary among both traits and species using Bayesian generalised linear mixed effects models implemented in the R package *MCMCglmm* version 2.3.4 (Hadfield, 2010). This package has the option of implementing a random phylogenetic effect (Hadfield & Nakagawa, 2010) and allows us to obtain posterior distributions of all fixed and random effects rather than point estimates and SEs as provided by restricted maximum likelihood (REML) approaches (Morrissey et al., 2014). Although the distributions of *h* ^2^ and *CV*_A_ are poorly understood (Wood et al., 2016), all models assumed Gaussian error distributions.

To test for a phylogenetic signal in *h* ^2^ and *CV*_A_, we built robust maximum clade credibility bird and mammal supertrees using *TreeAnnotator*, part of the BEAST version 1.10.4 package (Rambaut et al., 2014). These were based on 1000 randomly sampled trees from a pseudo‐posterior distribution of species‐level phylogenies (available at: https://vertlife.org/phylosubsets/) and based on Jetz et al. (2012) using the Hackett et al. (2008) backbone for birds and Upham et al. (2019) for mammals. Both trees were then imported and combined in R version 4.2.1 (R Core Team, 2020) using the packages *ape* version 5.6.2 (Paradis & Schliep, 2019), *phytools* version 1.2.0 (Revell, 2012) and *geiger* version 2.0.10 (Pennell et al., 2014). The tree was rooted at 315 million years based on the dating of *Archerpeton anthracos* and the origin of all amniotes (as recommended by Healy et al., 2014).

We fitted phylogenetic mixed models to both *h* ^2^ and *CV*_A_. Fixed effects included the *study method* (e.g. parent–offspring regression), *trait category* (behaviour, morphology, physiology, LRS, survival (i.e. fitness) and other life‐history traits) and the *sample size* and *study year* as covariates. Both *studies* and *species* were included as random effects. The *species* effect controlled for repeated species measures and estimated the level of interspecific variation. Phylogenetic relatedness was accounted for following Hadfield and Nakagawa (2010) and modelled assuming Brownian motion. Measurement error was incorporated by inserting the squared SE of estimates into the *mev* argument in *MCMCglmm()*.Thereby, this model provides both method‐ and trait‐standardised estimates of interspecific variation in *h* ^2^ and *CV*_A_ and method‐ and species‐standardised estimates of *h* ^2^ and C*V*_A_ for each trait category, while controlling for potential non‐species independence.

We used the default weakly informative parameter expanded priors set to *F*_1,1_ distributions (scale = 1000) for random effects, the Inverse‐Wishart distribution for the residual variance, and non‐informative priors for the fixed effects. We ran each model for 1,505,000 iterations, with a thinning interval of 1500 and a burn‐in of 5000. This ensured effective sample sizes greater than 1000. We evaluated model convergence and mixing based on the visual examination of trace plots (Hadfield, 2010). This inclusion of a measure of sampling error for *CV*_A_ in particular should help combat any scaling effects in the precision of the parameters (Garcia‐Gonzalez et al., 2012). Fixed effects with MCMC *p*‐values of less than .05 were judged to be statistically significant. Estimates for fixed and random effects are obtained from the mode of the posterior distribution, and all estimates are accompanied by their 95% credible intervals. To aid interpretation, variance components were also expressed as percentages of the total amount of variance (with 95% credible intervals) excluding measurement error. Figures were created using the packages *ggplot2* version 3.4.0 (Wickham, 2016) and *ggpubr* version 0.4.0 (Kassambara, 2020).

## 3 RESULTS

### 3.1 Heritability (*h* ^2^)

**FIGURE 1:**
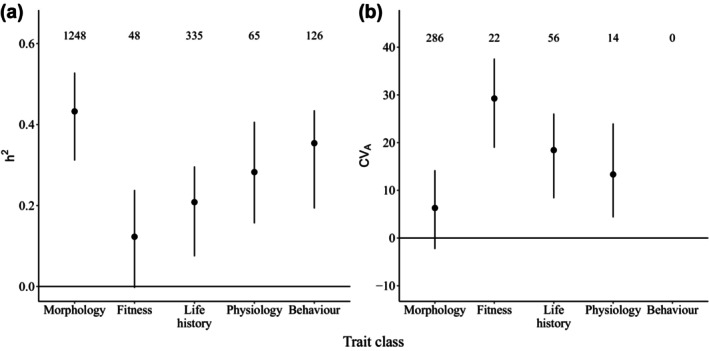
Variation in *h* ^2^ and *CV*_A_ among trait categories. Method‐standardised and species‐ and study‐independent estimates for (a) *h* ^2^ and (b) *CV*_A_ for each trait category, assuming they are predicted using a parent–offspring regression, using mean values for sample size and study year and controlling for species, study and phylogenetic non‐independence. Dots show the posterior mode of the predicted trait category estimate, with bars showing 95% credible intervals. The numbers show the number of estimates in the dataset for each trait category. No estimates of *CV*_A_ were available for behavioural traits.

**FIGURE 2:**
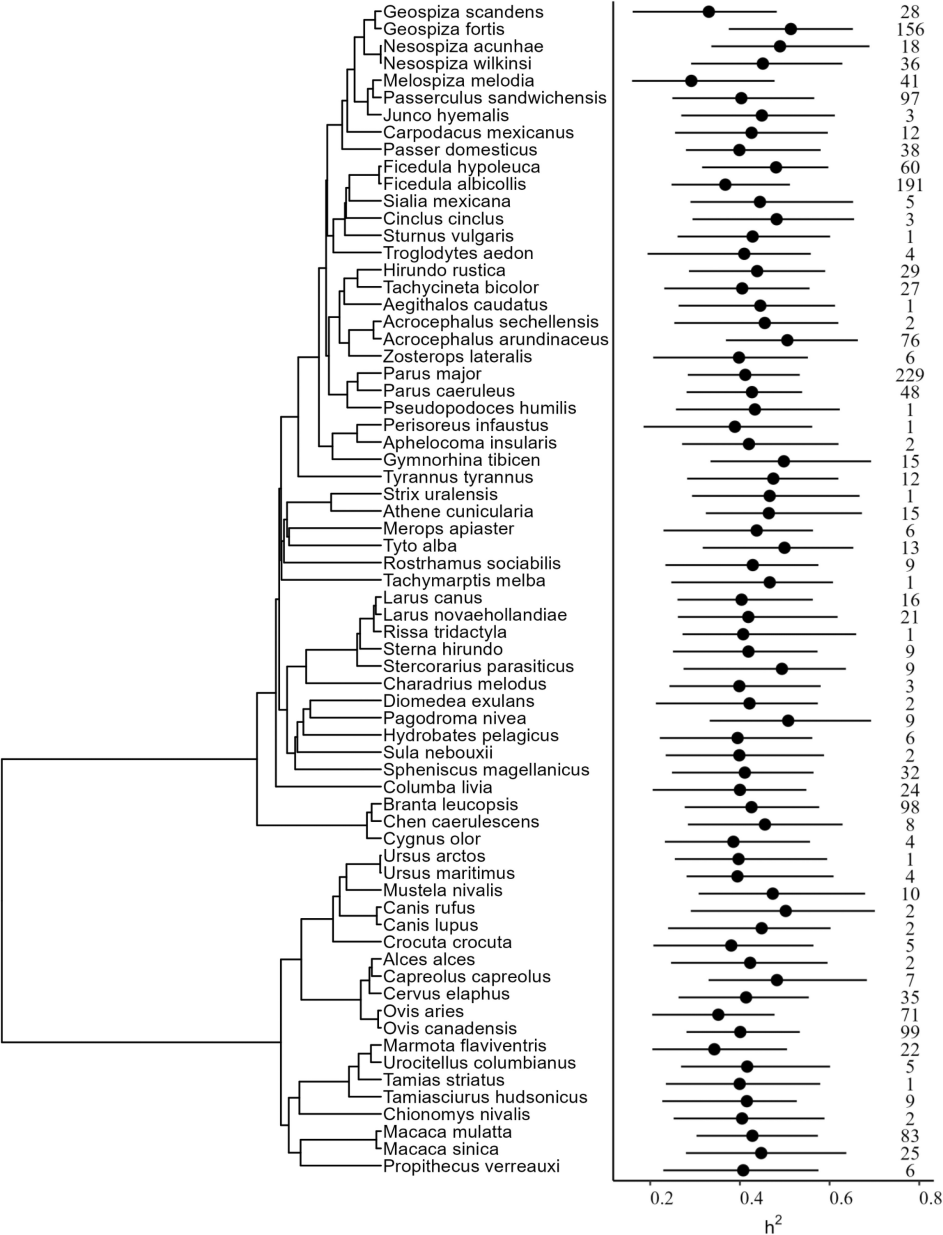
Standardised *h* ^2^ estimates for each species across phylogeny. Phylogenetic tree (left) of birds and mammals with species names and predicted *h* ^2^ estimates (right) from a MCMC linear mixed model for each of the 68 species. Estimates for each species were predicted for a parent–offspring regression, a morphological trait and using mean values for sample size and study year. Dots show the posterior modes, with bars showing 95% credible intervals. The numbers show the number of estimates for each species included in the analysis.

We analysed 1822 *h* ^2^ estimates from 214 studies and 68 different species of birds and mammals (49 and 19, respectively). Estimates were based on 5 (Møller, 1991) to 38,024 individual phenotypes (Garant et al., 2004) from studies published between 1974 and 2020. The overall mean *h* ^2^, incorporating measurement error, was estimated at 0.35 [95% CI 0.34, 0.37], and the variance in *h* ^2^ estimates was estimated at 0.06 [95% CI 0.05, 0.06]. There was a clear overrepresentation of *h* ^2^ estimates for morphological traits (*n* = 1248), with fitness (*n* = 48), life history (*n* = 335), physiology (*n* = 65) and behaviour (*n* = 126) traits being far less numerous (Table 1 and Figure 1a). The number of *h* ^2^ estimates per species ranged from 1 to 229 (Figure 2). There was also a clear bias in the number of estimates towards some species: The top five species with the most numerous estimates accounted for 42% of all estimates, and four out of these five species were birds (Great tit (*Parus major*): *n* = 229; Collared flycatcher (*Ficedula albicollis*): *n* = 191; Darwin's medium ground finch (*Geospiza fortis*): *n* = 156; Bighorn sheep (*Ovis canadensis*): *n* = 99; Barnacle goose (*Branta leucopsis*); *n* = 98).

**TABLE 1:** Output from the MCMC generalised linear mixed model (GLMM) with *h* ^2^ as the dependent variable.

Fixed effects	Posterior mode [95% CrIs]	*p*_MCMC_
(Intercept)	0.43 [0.31, 0.53]	<.001
Method, animal model (MCMC)	−0.01 [−0.06, 0.04]	.859
Method, animal model (REML)	−0.01 [−0.06, 0.06]	.971
**Method, full‐sib**	**0.24 [0.17, 0.31]**	**<.001**
Method, half‐sib	0.09 [−0.08, 0.32]	.252
**Method, grandparent–offspring regression**	**0.26 [0.07, 0.44]**	**.009**
**Trait category, fitness**	**−0.29 [−0.35, −0.25]**	**<.001**
**Trait category, life history**	**−0.22 [−0.26, −0.2]**	**<.001**
**Trait category, behaviour**	**−0.1 [−0.17, −0.04]**	**<.001**
**Trait category, physiology**	**−0.13 [−0.21, −0.07]**	**<.001**
**Study year**	**−8.42 × 10** ^**−3**^ **[−5.63 × 10** ^**−3**^ **, −3.07 × 10** ^**−3**^ **]**	**<.001**
**Sample size**	**−7.85 × 10** ^**−6**^ **[−1.55 × 10** ^**5**^ **, −7.99 × 10** ^**−7**^ **]**	**.033**

Random effects	Posterior mode [95% CrIs]	Percentage variance explained [95% CrIs]
Species	0.0054 [0.0012, 0.0134]	14.92% [3.48, 30.85]
Phylogeny	0.0001 [0.0000, 0.0214]	0.23% [0.00, 39.00]
Study	0.0153 [0.0111, 0.0211]	40.23% [23.34, 52.71]
Residual	0.0140 [0.0121, 0.0156]	34.27% [22.90, 45.09]

*Note*: Method and trait category were included as fixed categorical variables, and sample size and year of publication as fixed covariates. Sample size and study year were mean centred so that the intercept shows predicted values for estimates based on a parent–offspring regression for a morphological trait, a study published in 2002 and a sample size of 484. *Species, phylogeny* and *study* were included as random effects. For fixed effects, the posterior modes with 95% credible intervals and MCMC *p*‐values (*p*_MCMC_) are shown, with bold highlighting *p*_MCMC_ < .05. For random effects, the posterior modes and the variation explained (as a percentage) are shown with 95% credible intervals. The variation explained was calculated as the posterior estimates of the variance divided by the total of all the posterior estimates (excluding measurement error). The posterior mode and credible intervals were then extracted from this.

Estimates are lower in more recent studies and for larger sample sizes (Table 1). Furthermore, they are dependent on the estimation method used: full‐sib analyses and grandparent–offspring regressions provide larger estimates than the animal model (Bayesian or REML) and parent–offspring regressions (Table 1). We also found significant differences in *h* ^2^ estimates among trait types: conditioned upon other effects; morphology traits were estimated to have the highest *h* ^2^ (0.43 [95% CI 0.31, 0.53]), followed by physiology and behaviour traits having moderate *h* ^2^ (0.28 [95% CI 0.16, 0.41] and 0.35 [95% CI 0.19, 0.44], respectively; Figure 1a). Fitness traits had slightly lower *h* ^2^ than life‐history traits (0.12 [95% CI 0.00, 0.24] and 0.21 [95% CI 0.07, 0.30], respectively; Figure 1a).

**FIGURE 3:**
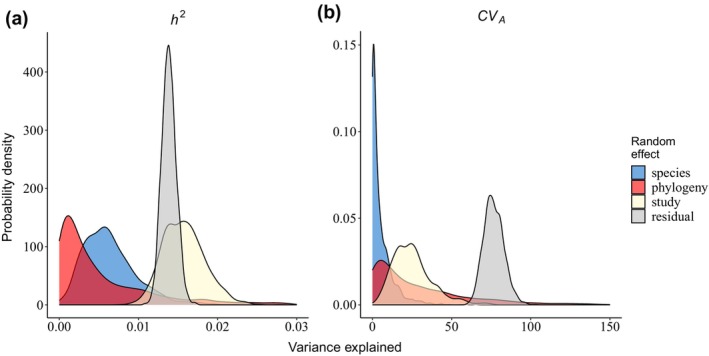
Variance explained by random effects. Posterior distributions of the variance in (a) *h* ^2^ and (b) *CV*_A_ explained by random effects from the MCMC generalised linear mixed models, conditioned on fixed effects. For posterior modes and 95% credible intervals, see Tables 1 and 2.

**FIGURE A1:**
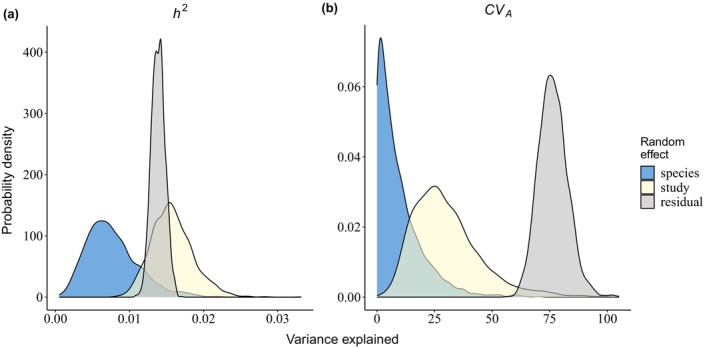
Variance explained by random effects in models without phylogenetic effect. Posterior distributions of the variance in (a) *h* ^2^ and (b) *CV*_A_ are explained by random effects from the MCMC generalised linear mixed models containing no phylogenetic effect. For posterior modes and 95% credible intervals, see Tables A1 and A4.

**FIGURE A2:**
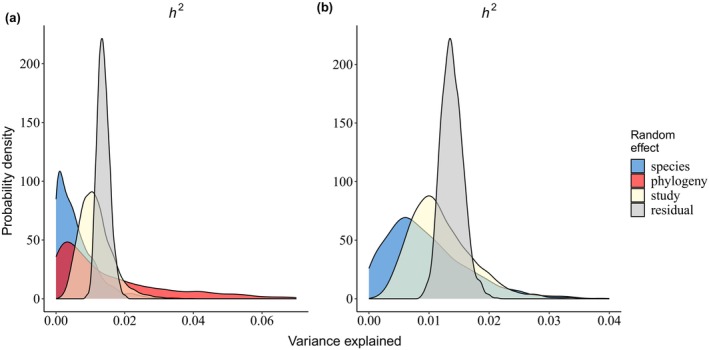
Variance in *h* ^2^ explained by random effects using data for which only *CV*_A_ estimates were available. Posterior distributions of the percentage of variance in *h* ^2^ explained by random effects from the MCMC generalised linear mixed models using only estimates for which *CV*_A_ estimates were available and (a) with and (b) without a phylogenetic effect included in the model, conditioned on fixed effects. For posterior modes and 95% credible intervals, see the text.

The variance in *h* ^2^ left unexplained by the fixed effects was 0.0348, of which 0.005 was explained by *species*, 0.015 by *study* and 0.014 remained unexplained (Table 1, Figure 3a). Although estimated with substantial uncertainty, *phylogeny* most likely explained little to no variation (0.001, Table 1). These estimates remained similar when the phylogenetic effect was removed from the model (Figure A1a and Table A1 in Appendix 1). However, when repeating the *h* ^2^ analysis using only estimates available for the *CV*_A_ analysis, we found that the variance explained by *species* was similar when the phylogenetic was excluded from the model (0.006, Table A2 and Figure A2b in Appendix 1) but decreased when the phylogenetic was included (0.0005, Table A3 and Figure A2a in Appendix 1), even though the variance explained by phylogeny was very small too (0.0001), suggesting there was a lack of power to disentangle these two effects. Other estimates remained consistent across all models.

Predicted method‐ and trait‐standardised estimates for each species are presented in Figure 2. Of all species, the Song sparrow (*Melospiza melodia*) had the lowest *h* ^2^ of 0.29 [95% CI 0.16–0.48] (for a parent–offspring method, morphological trait and using median values for sample size, study length and study year), whereas the Darwin's medium ground finch (*Geospiza fortis*) had the highest *h* ^2^ of 0.51 [95% CI 0.38–0.65] (Figure 2).

### 3.2 Coefficient of additive genetic variance ( *CV*_A_ )

With 378 estimates from 52 studies of 23 different species of birds and mammals, the dataset for *CV*_A_ was substantially smaller than for *h* ^2^. Estimates covered the years from 1989 to 2019, with the number of individual phenotypes on which estimates were based ranging from 19 (Larsson et al., 1997) to 38,024 (Garant et al., 2004). Morphological traits were most numerous (*n* = 286), with fewer life histories (*n* = 56), fitness (*n* = 22) and physiological traits (*n* = 14) (Figure 1b). As they are generally measured on a scale that lacks a natural zero, no estimates of *CV*_A_ were available for behavioural traits. The weighted mean and variance for *CV*_A_ were 7.33 [95% CI 6.19–8.49] and 145.62 [95% CI 123.83–165.69], respectively. The dataset contained *CV*_A_ estimates for 13 species of birds and 10 mammals. These ranged from 1 to 59 estimates per species.

*CV*_A_ estimates were not affected by study method, year or sample size (*p*_MCMC_‐values > .05, Table 2). Morphological traits had the lowest predicted *CV*_A_, and fitness traits had the highest (posterior mode = 6.30 [95% CI –2.30, 14.21] and posterior mode = 29.26 [95% CI 18.94, 37.59], respectively; Figure 1b). Life‐history traits had a lower *CV*_A_ than fitness traits (posterior mode = 18.42 [95% CI 8.35, 26.08]), with physiology traits having moderate *CV*_A_ estimates (posterior mode = 13.33 [95% CI 4.35, 24.00]; Figure 1b). Method‐standardised and species‐independent *CV*_A_ estimates for each trait category were predicted using a GLMM (Table 2) and are presented in Figure 1b.

**TABLE 2:** Output from the MCMC generalised linear mixed model (GLMM) with *CV*_A_ as the dependent variable.

Fixed effects	Posterior mode [95% CrIs]	*p*_MCMC_
(Intercept)	6.30 [−2.30, 14.21]	.148
Method, animal model (REML)	−0.48 [−4.45, 4.03]	.884
Method, animal model (MCMC)	−2.92 [−14.02, 5.18]	.457
Method, full‐sib	−0.40 [−12.23, 11.03]	.883
**Trait category, fitness**	**22.99 [18.61, 27.69]**	**<.001**
**Trait category, life history**	**11.74 [8.55, 14.57]**	**<.001**
**Trait category, physiology**	**7.37 [2.68, 15.04]**	**<.004**
Year	0.16 [−0.19, 0.48]	.453
Sample size	1.15 × 10^−4^ [−4.63 × 10^−4^, 5.77 × 10^−4^]	.825

Random effects	Posterior mode [95% CrIs]	Percentage variance explained [95% CrIs]
Species	0.62 [0.00, 105.4]	0.25% [0.00, 49.91]
Phylogeny	0.06 [0.00, 19.18]	0.03% [0.00, 14.31]
Study	23.92 [4.48, 52.22]	13.35% [3.15, 35.24]
Residual	73.00 [64.99, 88.85]	61.56% [31.67, 81.26]

*Note*: Estimation method and trait category were included as fixed categorical variables, and sample size and year of publication as fixed covariates. Sample size and study year were mean centred so that the intercept shows predicted value estimates based on a parent–offspring regression for a morphological trait, a study published in 2006 and a sample size of 768. *Species* and *studies* were included as random effects. For fixed effects, the posterior modes with 95% credible intervals and MCMC *p*‐values (*p*_MCMC_) are shown, with bold highlighting *p*_MCMC_ < .05. For random effects, the posterior modes and the variation explained (as a percentage) are shown with 95% credible intervals. The variation explained was calculated as the posterior estimates of the particular effect divided by the total of all the posterior estimates. The posterior mode and credible intervals were then extracted from this.

The variance left unexplained by the fixed effects was 97.60, of which 0.62 was explained by *species*, 23.92 by *study* and 73.00 remained unexplained by the random effects (Table 2, Figure 3b). In line with the small amount of interspecific variance, there was little evidence for a phylogenetic signal of 0.06 (Table 2, Figure 2b). These effects remained unchanged when the phylogenetic effect was removed from the models (Table A4 and Figure A1b in Appendix 1).

## 4 DISCUSSION

To better understand the mechanisms shaping additive genetic variances, we performed phylogenetic comparative analyses of *h* ^2^ and *CV*_A_ estimates to quantify interspecific variation in additive genetic variances in the wild. After accounting for within‐species differences (attributable to methodology and trait type), we found no interspecific variation in *CV*_A_ (<1%; Figure 2), but we did in *h* ^2^ estimates (of around 15%). However, the latter variation was not associated with the phylogeny. Based on these findings, we cautiously present the following conclusions, acknowledging the low power of some of these analyses.

The low but non‐zero level of interspecific variation in *h* ^2^ and the absence of interspecific variation in *CV*_A_ agree with previous analyses (Mittell et al., 2015; Postma, 2014). Interspecific variation in *h* ^2^ but not *CV*_A_ was unlikely to be due to a difference in power between the two analyses, as the difference persisted when the *h* ^2^ analysis was performed using only the estimates for which estimates for *CV*_A_ were available too and when we did not fit a phylogenetic effect (Figure A2 in Appendix 1). However, when the phylogenetic effect was included in this model, the variance explained by species was reduced, despite the phylogeny explaining no variation. This is suggestive of insufficient phylogenetic power in these models due to the smaller *CV*_A_ dataset (Tables A2 and A3, and Figure A2 in Appendix 1). In fact, the credible intervals for both *h* ^2^ and *CV*_A_ for the phylogenetic effect are large. Nevertheless, these estimates do broadly agree with previous findings (Dochtermann et al., 2019; Martinossi‐Allibert et al., 2017), although these analyses are likely to have been even more limited in terms of power. Clearly, future studies examining a phylogenetic effect on additive genetic variance should either wait for a considerable increase in the number of available estimates or broaden the scope of their study to include more taxa. None of the models (with or without a phylogenetic effect) revealed interspecific variation in *CV*_A_ (Table A4 and Figure A1b in Appendix 1). While together this suggests that interspecific variation in *h* ^2^ may be larger than it is in *CV*_A_, the 95% credible intervals for both variances overlap, and thus no statistically significant difference can be inferred (Gelman & Stern, 2006).

Bearing in mind this caveat, the finding of a larger interspecific variation in *h* ^2^ than *CV*_A_ would be in line with the level of environmental variance in traits being the driving factor in the interspecific variation in *h* ^2^. Following this logic, *h* ^2^ estimates for Soay sheep (*Ovis aries*) would be among the lowest of all species included in this analysis, not due to particularly low levels of additive genetic variance, even if this could be expected based on their unique demographic characterised by bottlenecks and isolation (Clutton‐Brock & Pemberton, 2004). Instead, they would be low because they inhabit a particularly variable environment and/or they respond particularly strongly to any environmental variation (Robinson et al., 2009; Wilson et al., 2006).

Aside from the possibility of non‐additive genetic effects (Mackay, 2014), which are likely to be small in wild populations regardless (Class & Brommer, 2020), *h* ^2^ between species may vary among species in a way that is similar to how environmentally induced variation (i.e. plasticity) drives variation in *h* ^2^ between trait types (Kruuk et al., 2000). For example, *h* ^2^ estimates may be lower in species exposed to unfavourable conditions, which could decrease *h* ^2^ through an increase in the importance of environmental factors (Charmantier & Garant, 2005; Gebhardt‐Henrich & Van Noordwijk, 1991). Future studies could test this hypothesis, provided they are able to measure the quality of the environment in a manner that allows for across species comparisons.

Although we currently do not have a good understanding of the role of (genetic or environmentally induced) variation in trait *means* in shaping variation in *CV*_A_ among species, a more definitive test would be to compare the interspecific variation in *CV*_A_ and *CV*_E_ (the mean‐scale non‐additive genetic and/or environmental variance). However, estimates of *CV*_E_ are currently rarely published, and often without the estimates of precision that enable a formal meta‐analysis. Overall, it is likely that further comparative studies of these trends in wild populations are limited by the availability of standardised estimates of *V*_A_. Not only are estimates extremely biased towards specific species with long‐running field studies, but *CV*_A_ still remains far less widely reported than *h* ^2^, despite little difference in the difficulty of reporting the latter. Given the well‐known difficulties of interpreting estimates of *h* ^2^ without knowledge of the absolute levels of additive genetic and environmental variance, reporting both metrics together, whenever possible, would be an improvement to the literature. Nevertheless, because coefficients of variation are only meaningful for a subset of traits (as discussed below), sample sizes will always be smaller than for estimates of heritability.

The range of sample sizes across both trait types and species highlights the substantial biases in the literature. First, the vast majority of traits were morphology traits, and while the sample sizes for fitness and life‐history traits were enough to confirm previous findings of the variation in *V*_A_ among trait types (e.g. Mittell et al., 2015; Mousseau & Roff, 1987; Postma, 2014), for physiological and behavioural traits, the patterns are less clear due to data limitations. While technological advancements mean that physiological traits are now more often measured in wild populations (e.g. (Béziers et al., 2019)), the *CV*_A_ of behavioural traits is generally non‐calculable as commonly reported behaviours, like boldness, do not fall on a true ratio scale (Hansen et al., 2011), limiting our understanding of the evolvability of these important traits. Despite its obvious advantages, the fact that *CV*_A_ can only be calculated for non‐random subsets of traits is an important limitation when it comes to comparative analyses of additive genetic variance. Second, the vast majority of estimates were restricted to a limited number of species and populations. While this highlights the importance of long‐term, individual‐based datasets (Clutton‐Brock & Sheldon, 2010), the bias in the literature towards some species (birds in particular) is a concern. The bias in methods is less problematic, with the literature moving towards the use of animal models, and our results confirm that they give smaller estimates of *h* ^2^, presumably because they are less likely to be biased by confounding non‐genetic effects (Mittell et al., 2015; Postma, 2014; Postma & Charmantier, 2007; Wood et al., 2016).

How *h* ^2^ and *CV*_A_ vary across different methods and traits is relatively clear, with consistent findings between studies, but we still lack an understanding of what shapes estimates of coefficients of additive genetic variance between species. We show here that *h* ^2^ varies between species but that this appears not to relate to phylogeny. However, interpretation of our results is hampered by limited statistical power, in particular with respect to our analysis of *CV*_A_. This also meant that although our interspecific variation in *h* ^2^ was higher than that of *CV*_A_ (which was approximately zero), we were not able to demonstrate a statistically significant difference in the amount of interspecific variation in both measures of *V*_A_. There is therefore a need to move beyond this approach if we are to better understand the factors shaping the coefficients of additive genetic variance. First, understanding how estimates vary within a species between populations (e.g. Martínez‐Padilla et al. (2017); Pennington et al. (2021); Volis et al. (2014)) could be a useful avenue and perhaps an important prerequisite for understanding interspecific variation. Related to this, it should be noted that in this study we gathered data measured at the population level and aggregated them by species, meaning interpopulation differences could mask or drive interspecific variation. Second, there may be a need to move beyond univariate measures of additive genetic variance, as traits do not exist in isolation (Nicolaus et al., 2016; Poissant et al., 2016) and selection on one trait often affects the evolution of others (Gould & Lewontin, 1979; Lande, 1979; Phillips & Arnold, 1989). Although broad trends are yet to be determined, our hope is that in future years, when the number of estimates available is (even) larger and—most importantly—more diverse, we will be able to understand how additive genetic variance varies across species and the mechanisms responsible for this variation.

## AUTHOR CONTRIBUTIONS

**Euan A. Young:** Conceptualization (equal); data curation (equal); formal analysis (lead); investigation (equal); methodology (equal); resources (equal); software (equal); validation (equal); visualization (equal); writing – original draft (lead); writing – review and editing (equal). **Erik Postma:** Conceptualization (equal); data curation (equal); formal analysis (supporting); funding acquisition (lead); investigation (equal); methodology (equal); project administration (lead); resources (equal); software (equal); supervision (lead); validation (equal); visualization (equal); writing – original draft (supporting); writing – review and editing (equal).

## FUNDING INFORMATION

Swiss National Science Foundation grants 141110 and 159462.

## CONFLICT OF INTEREST STATEMENT

The authors declare no conflicts of interest.

## Data Availability

All code and data are deposited at: https://doi.org/10.34894/RIVFHW.

## APPENDIX 1

**TABLE A1:** Output from the MCMC generalised linear mixed model (GLMM) with *h* ^2^ as the dependent variable for all *h* ^2^ estimates, but no phylogenetic effect included.

Fixed effects	Posterior mode [95% CrIs]	*p*_MCMC_
(Intercept)	**0.44 [0.39, 0.49]**	**<.001**
Method, animal model (REML)	−0.01 [−0.06, 0.05]	.771
Method, animal model (MCMC)	0.01 [−0.07, 0.06]	.956
**Method, full‐sib**	**0.23 [0.16, 0.30]**	**<.001**
Method, half‐sib	0.07 [−0.09, 0.29]	.271
**Method, grandparent–offspring regression**	**0.21 [0.03, 0.43]**	**<.023**
**Trait category, fitness**	**−0.30 [−0.35, −0.25]**	**<.001**
**Trait category, life history**	**−0.23 [−0.26, −0.20]**	**<.001**
**Trait category, behaviour**	**−0.10 [−0.17, −0.05]**	**<.001**
**Trait category, physiology**	**−0.13 [−0.21, −0.07]**	**<.001**
**Study year**	**5.68 × 10** ^**−3**^ **[−8.51 × 10** ^**−3**^ **, −3.05 × 10** ^**−3**^ **]**	**<.001**
**Sample size**	**−7.97 × 10** ^**−6**^ **[−1.54 × 10** ^**−5**^ **, 6.82 × 10** ^**−9**^ **]**	**.044**

Random effects	Posterior mode [95% CrIs]	Percentage variance explained [95% CrIs]
Species	0.0053 [0.0019, 0.0150]	16.81% [5.85, 35.00]
Study	0.0152 [0.0104, 0.0213]	40.49% [30.45, 55.68]
Residual	0.0136 [0.0122, 0.0156]	35.09% [29.69, 45.76]

*Note*: Method and trait category were included as fixed categorical variables, and sample size and year of publication as fixed covariates. Sample size and study year were mean centred so that the intercept shows predicted values for parent–offspring regression method, morphological traits, study year 2002 and a sample size of 484. *Species* and *studies* were included as random effects. For fixed effects, the posterior modes with 95% credible intervals and MCMC *p*‐values (*p*_MCMC_) are shown, with bold highlighting *p*_MCMC_ < .05. For random effects, the posterior modes and the variation explained (as a percentage) are shown with 95% credible intervals. The variation explained was calculated as the posterior estimates of the particular effect divided by the total of all the posterior estimates. The posterior mode and credible intervals were then extracted from this.

**TABLE A2:** Output from the MCMC generalised linear mixed model (GLMM) with *h* ^2^ as the dependent variable using only data for which *CV*_A_ estimates were available with no phylogenetic effect included.

Fixed effects	Posterior mode [95% CrIs]	*p*_MCMC_
**(Intercept)**	0.44 [0.33, 0.52]	.001
Method, animal model (REML)	−0.07 [−0.14, 0.04]	.235
Method, animal model (MCMC)	−0.1 [−0.26, 0.15]	.545
**Method, full‐sib**	**0.36 [0.04, 0.74]**	**.031**
**Trait category, fitness**	**−0.31 [−0.39, −0.22]**	**.001**
**Trait category, life history**	**−0.19 [−0.26, −0.13]**	**.001**
Trait category, physiology	−0.11 [−0.22, 0]	.053
Year	−6.83 × 10^−6^ [−1.14 × 10^−2^, 2.50 × 10^−3^]	.201
Sample size	−6.83 × 10^−6^ [−1.61 × 10^−5^, 1.71 × 10^−6^]	.099

Random effects	Posterior mode [95% CrIs]	Percentage variance explained [95% CrIs]
Species	0.0063 [0, 0.0228]	15.77% [0, 50.03]
Study	0.0098 [0.0033, 0.0223]	32.5% [10.29, 59.21]
Residual	0.0132 [0.0106, 0.0176]	37.65% [24.39, 55.95]

*Note*: Method and trait category were included as fixed categorical variables, and sample size and year of publication as fixed covariates. Sample size and study year were mean centred so that the intercept shows predicted values for parent–offspring regression method, morphological traits, study year 2006 and a sample size of 768. *Species* and *studies* were included as random effects. For fixed effects, the posterior modes with 95% credible intervals and MCMC *p*‐values (*p*_MCMC_) are shown, with bold highlighting *p*_MCMC_ < .05. For random effects, the posterior modes and the variation explained (as a percentage) are shown with 95% credible intervals. The variation explained was calculated as the posterior estimates of the particular effect divided by the total of all the posterior estimates. The posterior mode and credible intervals were then extracted from this.

**TABLE A3:** Output from the MCMC generalised linear mixed model (GLMM) with *h* ^2^ as the dependent variable using only data for which *CV*_A_ estimates were available, with the phylogenetic effect included.

Fixed effects	Posterior mode [95% CrIs]	*p*_MCMC_
**(Intercept)**	**0.42 [0.20, 0.62]**	**.005**
Method, animal model (REML)	−0.06 [−0.15, 0.04]	.307
Method, animal model (MCMC)	−0.06 [−0.25, 0.13]	.657
**Method, full‐sib**	**0.45 [0.03, 0.72]**	**.035**
**Trait category, fitness**	**−0.31 [−0.36, −0.21]**	**.001**
**Trait category, life history**	**−0.2 [−0.25, −0.13]**	**.001**
Trait category, physiology	−0.1 [−0.23, −0.01]	.056
Year	−4.31 × 10^−3^ [−1.26 × 10^−2^, 1.87 × 10^−3^]	.133
Sample size	−7.83 × 10^−6^ [−1.60 × 10^−5^, 7.44 × 10^−6^]	.067

Random effects	Posterior mode [95% CrIs]	Percentage variance explained [95% CrIs]
Species	0.0005 [0.0000, 0.0700]	0.38% [0.00, 72.23]
Phylogeny	0.0001 [0.0000, 0.0167]	0.18% [0.00, 36.13]
Study	0.0101 [0.0031, 0.0209]	20.62% [5.45, 49.54]
Residual	0.0137 [0.0105, 0.0174]	31.57% [10.53, 52.75]

*Note*: Method and trait category were included as fixed categorical variables, and sample size and year of publication as fixed covariates. Sample size and study year were mean centred so that the intercept shows predicted values for parent–offspring regression method, morphological traits, study year 2006 and a sample size of 768. *Species* and *studies* were included as random effects. For fixed effects, the posterior modes with 95% credible intervals and MCMC *p*‐values (*p*_MCMC_) are shown, with bold highlighting *p*_MCMC_ < .05. For random effects, the posterior modes and the variation explained (as a percentage) are shown with 95% credible intervals. The variation explained was calculated as the posterior estimates of the particular effect divided by the total of all the posterior estimates. The posterior mode and credible intervals were then extracted from this.

**TABLE A4:** Output from the MCMC generalised linear mixed model (GLMM) with *CV*_A_ as the dependent variable and with no phylogenetic effect included.

Fixed effects	Posterior mode [95% CrIs]	*p*_MCMC_
**(Intercept)**	**5.56 [0.75, 9.64]**	**.019**
Method, animal model (REML)	0.26 [−4.72, 4.04]	.875
Method, animal model (MCMC)	−2.40 [−14.01, 5.49]	.439
Method, full‐sib	−1.62 [−12.44, 9.17]	.833
**Trait category, fitness**	**22.72 [18.62, 27.49]**	**<.001**
**Trait category, life history**	**10.93 [8.54, 14.72]**	**<.001**
**Trait category, physiology**	**8.96 [2.86, 14.88]**	**.007**
Year	0.03 [−0.24, 0.45]	.576
Sample size	−2.00 × 10^−5^ [−4.98 × 10^−4^, 5.60 × 10^−4^]	.984

Random effects	Posterior mode [95% CrIs]	Percentage variance explained [95% CrIs]
Species	0.17 [0.00, 28.73]	0.12% [0.00, 21.73]
Study	25.98 [7.72, 61.18]	25.20% [9.26, 44.72]
Residual	74.03 [63.67, 87.89]	70.77% [47.23, 84.82]

*Note*: Method and trait category were included as fixed categorical variables, and sample size and year of publication as fixed covariates. Sample size and study year were mean centred so that the intercept shows predicted values for parent–offspring regression method, morphological traits, study year 2006 and a sample size of 768. *Species* and *studies* were included as random effects. For fixed effects, the posterior modes with 95% credible intervals and MCMC *p*‐values (*p*_MCMC_) are shown, with bold highlighting *p*_MCMC_ < .05. For random effects, the posterior modes and the variation explained (as a percentage) are shown with 95% credible intervals. The variation explained was calculated as the posterior mode of the effect divided by the total of all the posterior modes.
